# Influence of Grinding Aids on the Grinding Performance and Rheological Properties of Cementitious Systems

**DOI:** 10.3390/ma17215328

**Published:** 2024-10-31

**Authors:** Yahya Kaya, Hatice Gizem Şahin, Naz Mardani, Ali Mardani

**Affiliations:** 1 Department of Civil Engineering, Bursa Uludag University, Bursa 16059, Turkey; 512126007@ogr.uludag.edu.tr (Y.K.); 532026001@ogr.uludag.edu.tr (H.G.Ş.); 2 Department of Mathematics Education, Bursa Uludag University, Bursa 16059, Turkey; nazmardani@uludag.edu.tr

**Keywords:** grinding aids, polycarboxylate ether-based water-reducing admixture, grinding efficiency, rheological property, thixotropic behavior, GA–PCE compatibility

## Abstract

The cement industry is of great importance in terms of raw materials consumed, energy consumed, and greenhouse gases emitted. Grinding aids (GA) are used to reduce energy consumption and costs, as well as to reduce the amount of CO_2_ released into the environment. In this study, the effect of GA-polycarboxylate ether-based water-reducing admixture (PCE) compatibility on some fresh, rheological and hardened state properties of cementitious systems was investigated. In order to investigate the rheological properties and thixotropic behavior of the mixtures, a total of 51 cement paste mixtures were prepared, containing 4 different types (molasses, MEG, DEA and ethanol) and ratios (0.025, 0.05, 0.75 and 0.1) of GAs and 2 different ratios (0.08% and 0.16%) of PCE in addition to the control mixture. In addition, the effect of the used GAs on the grinding efficiency and compressive strength value was investigated. Additionally, the predictability of the type of GA, dosage and cure time using the Taguchi method was investigated. It was determined that the highest grinding performance was obtained in mixtures containing MEG. It was determined that in cement paste mixtures containing GAs, the dynamic yield stress and viscosity values generally decrease with the increase in PCE usage rate up to a certain value, and these values may increase if the PCE usage increases further. It was determined that such behavior is not present in cement paste mixtures containing GAs and that the structural build-up value of the mixtures generally increases with the increase in the PCE admixture usage rate. It was determined that the use of GAs had a positive effect on 28-day compressive strength.

## 1. Introduction

Greenhouse gas emissions, air pollution and climate change are among the leading factors that cause global problems [1,2,3,4,5]. In the solution process of these problems, issues such as developing alternatives to products that cause CO_2_ emissions during production, renewable energy sources and energy efficiency have gained importance [6,7]. The cement industry causes great harm to the environment in terms of energy, raw material consumption and CO_2_ emissions. The amount of electricity spent in the cement industry constitutes 2% of the electricity used worldwide and 5% of the electricity used in the industry. However, it accounts for 5–7% of global CO_2_ emissions [8]. Approximately 1.2 tons of raw materials and 130 kWh of energy are consumed to produce one ton of cement, resulting in approximately 1 ton of CO_2_ [9,10]. In cement production, approximately 35% of the energy consumed is spent in the clinker grinding phase. In addition, a significant portion of the energy consumed in the grinding phase is wasted by converting into heat, sound and vibration. As a result of studies conducted to reduce both the energy consumption and cost and the amount of CO_2_ released into the environment, the use of grinding aids (GA) has come to the fore [11,12,13].

GAs adsorb on the surfaces of the grains with the help of the highly polar functional groups they contain (-OH, -NH_2_, -COOR, -SO_3_, etc.) [14,15]. Adsorbed GAs neutralize the electrical charges on the surface, preventing the cracks formed in the clinker from closing, particles from coming together and sticking to the mill surface and/or balls [15,16]. Thus, grinding efficiency is ensured. It is known that cement grains produced using GAs contain finer cement particles despite having the same Blaine fineness value. In addition, cements produced using GAs and cements produced without GAs create differences in important properties such as surface energy [17]. This situation significantly affects the hydration reactions of cement, cement–admixture compatibility and rheological properties of cement pastes.

Plank et al. [18] emphasized that GAs have a similar mechanism to polycarboxylate ether-based water-reducing admixtures (PCE) in terms of adsorption onto the cement grain. Therefore, the adsorption of PCE onto the cement grain may be negatively affected in systems containing GAs. It was stated that in case of binder–admixture incompatibility, negative effects such as rapid or slow setting [10], segregation [19], loss of consistency and increased shrinkage [19,20,21] may occur in cementitious systems. Some of these negativities may be due to their binding properties, while others may be due to admixture properties. Since the used GAs affect the cement properties, the cement–PCE compatibility of the cements in which GAs are used during production should be examined comprehensively. The effects on both the cement surface properties and hydration reactions vary depending on the type, dosage and chemical content of the GAs used [22,23,24,25]. Some studies examining the compatibility of cements produced with GAs with PCE are summarized below.

In a study conducted by Sun et al. [26], the compatibility of cements produced using 0.01, 0.02, 0.03 and 0.04% glycerin-type GAs with PCE was examined. It was observed that the flow performance of PCE was higher when the Ga dosage was less than 0.02%. It was determined that PCE was adsorbed at a lower rate in cements containing more than 0.02% GA. In another study, the effect of the use of GAs on the surface energy of the cement grain was investigated by Prziwara and Kwade [27]. On the one hand, it was stated that GAs adsorbed on the cement grain and caused the surface energy to decrease. On the other hand, it was emphasized that the cements produced using GAs may have higher surface energy compared to the grains without GAs due to the fact that they consist of smaller grains. This situation revealed that there are different opinions about the adsorption of PCE to cements containing GAs. It was stated by Karakuzu et al. [28] that PCE adsorption is seriously affected by the high positive charge density of the cement surface. It was emphasized by Sun et al. [26] that the cement surface load density can vary depending on the type of GA and the usage rate. Accordingly, the degree of adsorption of PCE to cement varies in cements containing GAs. As understood from the literature, it is necessary to examine the PCE compatibility of cementitious systems in the presence of GAs. It was reported by Mardani-Aghabaglou et al. [10] that the determination of rheological parameters of cementitious systems is one of the most reliable methods in terms of examining cement–admixture compatibility. However, studies examining the effect of cements produced with GAs on the rheological properties of cementitious systems are very limited.

With the Taguchi method, variables caused by uncontrollable factors that are not taken into account by traditional experimental design can be controlled [29]. In this method, the objective function values are converted to the signal-to-noise (S/N) ratio to measure the performance characteristics of the levels of control factors against the factors in question [29]. The S/N ratio is defined as the desired signal ratio for the undesired random noise value and shows the quality characteristics of the experimental data [30]. In this direction, it is known that the Taguchi method is used in experiments that require excessive energy and time.

In this study, GA–PCE compatibility was investigated in terms of rheological properties and thixotropic behavior of cement pastes. While rheological properties of mixtures were investigated with dynamic yield stress and viscosity values, thixotropic behavior was interpreted with structural build-up (A_thix_) behavior. The effect of used GAs on grinding efficiency and compressive strength values of mixtures was also investigated. In addition, the predictability of GA type, dosage and curing time was investigated with the Taguchi method.

## 2. Materials and Methods

### 2.1. Materials

The cements produced within the scope of this study were obtained by grinding 96% clinker and 4% gypsum in a laboratory-type ball mill until the target Blaine fineness value (3900 ± 100 cm^2^/g) was reached. The cements obtained are CEM I 42.5R-type cements in accordance with TS EN 197-1 Standard [31]. The chemical properties of clinker and gypsum given by the company are shown in Table 1.

Some properties of the GAs and PCE used in this study are shown in Table 2 and Table 3, respectively.

In the production of mortar mixtures, washed river sand aggregate with a grain diameter of 0–4 mm, in accordance with TS EN 196-1 Standard [32], was used. The saturated surface dry specific gravity of the aggregate according to TS EN 1097-6 Standard [33] was measured as 2.64 and its water absorption capacity as 1.2%.

### 2.2. Preparation of Mixtures and the Test Method

#### 2.2.1. Preparation of Mixtures

In order to determine the rheological properties and thixotropic behavior, the w/c ratio was kept constant as 0.35 in the produced paste mixtures. For this purpose, in addition to the control mixture, a total of 51 cement paste mixtures containing 4 different types (molasses, MEG, DEA and ethanol) and ratios (0.025, 0.05, 0.75 and 0.1) of GAs and 2 different ratios (0.08% and 0.16%) of PCE were prepared. The mixtures were named according to the type of GA, GA usage rate and PCE usage rate. For example, the mixture containing 0.050% ethanol and 0.08% PCE was named as 0.05 ethanol 0.08%. The rheological properties of the prepared mixtures were evaluated in terms of dynamic yield stress and viscosity parameters and thixotropic properties were evaluated in terms of structural build-up (A_thix_) development.

Within the scope of this study, mortar mixtures were produced in accordance with ASTM C109 Standard [34]. In determining the PCE requirements for the target flow value of the mixtures, the water/binder ratio, sand/binder ratio and target flow value were kept constant as 0.485, 2.75 and 190 ± 20 mm, respectively. All mixtures were prepared in a Hobart mixer.

#### 2.2.2. The Test Method

##### The Grinding Process

The clinker grinding process was carried out in a Bond-type laboratory-type mill with a 1.5 kW motor power and a capacity of 5 kg located in the Bursa Uludag University Construction Materials Laboratory. The Blaine fineness value of the obtained cements was determined by an automatic Blaine measuring device, and the particle size distributions were determined by a Malvern/Zetasizer Nano ZSP brand particle size determination device (Malvern, UK). In the grinding processes, the Blaine fineness value was measured after 30 min and 50 min of grinding. In addition, the number of revolutions applied until the target Blaine fineness value of 3900 ± 100 cm^2^/g was reached was determined. The energy consumed was determined based on the number of revolutions applied. The energy consumed in the production of cements was calculated according to Equation (1).

Eg = (220 × Tö × A × 1000)/(m × Tg)(1)

Here, Eg is the grinding energy (kWh/ton), Tö is the grinding time (hour), A is ampere, m is the mass of ground material (kg) and Tg is the mill factor (constantly taken as 4 from the grinding mill manufacturer). Optimum grinding conditions were determined based on the energy consumed.

##### The Rheology Test

**Figure 1:**
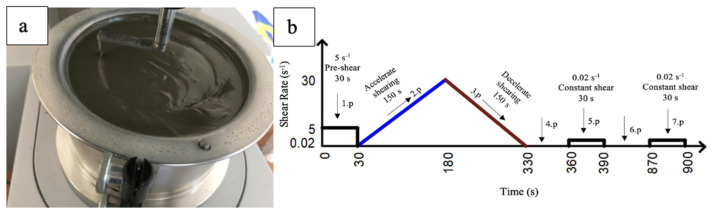
(**a**) Measuring device and (**b**) rheological measurement process.

The rheological properties and structural recovery development of the mixtures produced within the scope of this study were examined. In the scope of this study, an MCR52-Anton Paar rheometer (Istanbul, Turkey) with a ball diameter of 8 mm was used (Figure 1a). The ambient temperature was kept constant at 20 ± 2 °C during the measurement. The applied rheological measurement process is shown in Figure 1b.

**Figure 2:**
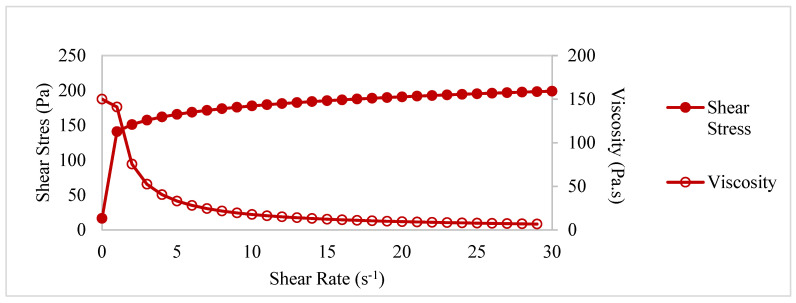
Shear stress–shear rate and viscosity–deformation rate graphs of control mixture.

The rheological properties of the mixtures (yield stress and final viscosity value) were obtained by drawing shear stress–shear rate and viscosity–shear rate graphs for each mixture, using the Herschel–Buckley model shown in Equation (2) on the raw data determined in the 3rd period. In Figure 2, as an example, the shear stress–shear rate and viscosity–deformation rate graphs of control mixture produced within the scope of this study are shown.

(2) $$\tau =\tau_{0}+b\dot{{Ɣ}^{p}}$$

Here, τ is the shear stress (Pa), $\tau_{0}$ is the yield stress (Pa), b is the Herschel–Bulkley consistency coefficient, $\dot{Ɣ\;}$ is the shear rate (s^−1^) and p is the Herschel–Bulkley index. 

The rheological measurement method consists of 7 periods. The shear rate and durations to which the mixtures are exposed in each period are shown in Figure 1b. The dynamic yield stress and viscosity values of the mixtures were determined using the 3rd period data. The maximum shear stress obtained from the 5th and 7th periods was selected as the static yield stress.

During the static shear test, the deformation rate to which the cementitious systems are exposed is very low and the mixture is not disturbed at all. In this case, only structural recovery is present in the mixtures. The structural recovery development (A_thix_) was determined using the static yield stresses obtained as a result of this test. The value in question was calculated with the help of Equation (3).

(3) $$A_{thix}=\frac{\tau_{s,f}-\tau_{s,i}}{t_{d}}$$

Here, $A_{thix}$ represents the structural recovery development (Pa/s), $\tau_{s,f}$ represents the static yield stress value (Pa) obtained from the 7th period, $\tau_{s,i}$ represents the static yield stress value (Pa) obtained from the 5th period, and $t_{d}$ represents the waiting time (s).

##### Flow and Compressive Strength

The flow value was determined in accordance with ASTM C1437 [35] and the compressive strength values in the prepared mixtures were determined according to ASTM C109 Standards.

##### Design of Experiments with the Taguchi Method 

Within the scope of this study, ANOVA and the Taguchi method were applied to determine the optimum combination of grinding parameters. In the Taguchi method, nominal best, largest best, smallest best methods depending on the characteristic type are used to calculate signal-to-noise (S/N) ratios [29]. In this study, the “biggest is best” as shown in Equation (4) proposed by Mandal et al. [36] is used as the objective function.

S/N = −10×log(Σ(1/Yi^2^)/n) (4)

where Yi is the data observed in the i-th experiment and n is the number of observations of the experiment.

In this study, the control factors were selected as GA type, GA dosage and curing time. The levels of the selected factors are shown in Table 4. In order to determine the optimum compressive strength value and analyze the effects of the factor parameters, the most appropriate orthogonal array was selected as L16(4^2 2^1). The L16 mixed orthogonal array shown in Table 5 was used to analyze the experimental results.

## 3. Experimental Results and Discussion

### 3.1. Grinding Efficiency

**Figure 3:**
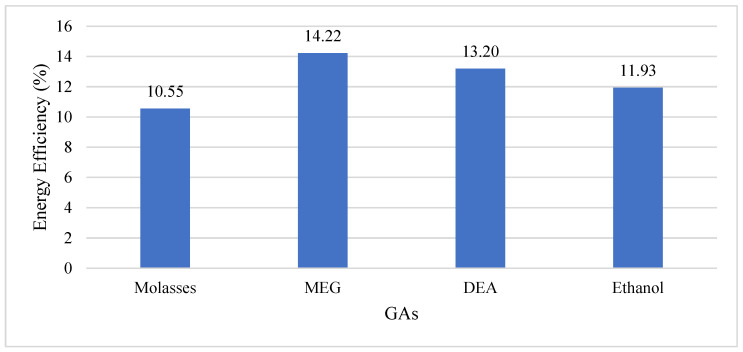
Average energy efficiency of Gas.

**Figure 4:**
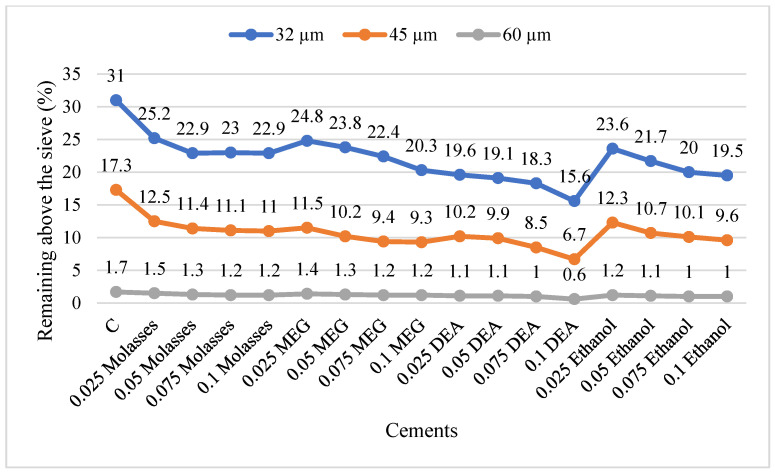
Particle size distributions of cements.

The Blaine fineness values of the cements with and without GAs after 30 min and 50 min grinding and the amount of energy consumed for the target Blaine fineness value are given in Table 6. In order to better understand the grinding efficiency of the GA types, the average grinding efficiencies are given in Figure 3 and the particle size distributions of the cements produced are given in Figure 4.

Regardless of the use of GAs, the fineness value of the cements increased as expected with the increase in grinding time from 30 min to 50 min. When the cements subjected to the 30 min grinding process were analyzed, it was found that the lowest Blaine fineness value belonged to the control cement without GAs. Compared to the control cement, cements containing molasses, MEG, DEA and ethanol-type GAs were measured to have 6-15-10% and 9% higher Blaine fineness values after 25 min of grinding, respectively. Among the GA types, the most successful performance on the early grinding time was observed with the glycol-based admixture, while the lowest performance was obtained with molasses admixture. Similarly, it was reported by Assaad et al. [37] that the performance of glycol-based admixtures was high at early grinding time. It was found that there was no significant increase in grinding efficiency with the increase in the rate of GA utilization regardless of the type of GA. It was found that the highest performance was obtained in molasses, MEG and ethanol types at 0.075% and in DEA at 0.025% of GA.

When the cements subjected to the 50 min grinding process were analyzed, it was found that the control cement without GAs had the lowest Blaine fineness value. It was determined that the use of molasses, MEG, DEA and ethanol increased the Blaine fineness values of the cements by 7-15-11% and 10%, respectively. After 30 min grinding, it was seen that the admixture performances continue similarly at 50 min grinding. It was determined that no significant pattern was observed with the increase in the rate of GA use.

While 47.2 kWh/ton of energy was consumed to achieve the target Blaine fineness of 4100 ± 100 cm^2^/g in control cement, the average energy consumed in cements containing molasses, MEG, DEA and ethanol was 42.22, 40.49, 40.97 and 41.57 kWh/ton, respectively (Figure 3). It was found that the highest grinding performance was achieved by using MEG with an energy efficiency of about 14.22% compared to the control cement. The lowest grinding performance was obtained when molasses admixture was used with 10.55% compared to the control cement (Figure 3). 

By adsorbing on the surface of the particles, GAs reduce the electrical charges generated by friction during grinding and the surface energies of the particles, which increase with the decrease in the size of the particles [38]. It has been emphasized by researchers that the dispersion effect of GAs adsorbed on the particle surface is related to the changing surface properties of the particles such as surface energy, zeta potential, and flocculation energy [39,40]. Since GAs adsorb on particles with the help of hydroxyl groups (-OH), the number of hydroxyl groups they have also affects their performance [14]. As can be seen from Table 2, the admixtures with the highest grinding performance have 2 hydroxyl groups in the molecular formula. In addition, Vieira and Peres [41] revealed in their study that the pH of the admixture used can have an effect on the grinding performance. In this study, it was observed that grinding efficiency increased with increasing pH. This indicates that the high grinding performance of DEA and MEG admixtures may also be related to the high pH value (Table 2).

It was found that the optimum grinding performance was observed for all GAs, regardless of the type of GA, when 0.075% SCT was used. In the literature, there are studies showing that the GA dosage reduces the efficiency after a certain point [42]. This is thought to be related to the powder fluidity of cement grains produced with GAs. When more than a certain amount of GA is used, cement grains may show sliding behavior at the collision point of the balls and cause a decrease in grinding performance [29].

Particle size distributions of cements with target Blaine fineness values are shown in Figure 4.

Since the particle size distribution of cements with the same Blaine fineness value may differ, it is known that Blaine fineness is not sufficient as a precise fineness criterion [42]. For this reason, the effect of the use of GAs on the particle size distribution was investigated. As can be seen from Figure 4, it was observed that the particle size distribution of the cements narrowed and a finer size range was obtained with the use of GAs and the increase in the utilization rate, regardless of the type of GA. In the studies conducted in the literature [15,42,43,44,45], it has been explained that cements obtained by using GAs result in narrower particle size distribution and smoother granular product formation compared to cements obtained without GAs. Accordingly, it was emphasized that the fine particle ratio increased. In grinding processes without GAs, the cement particles are agglomerated and coated on the balls, making the size reduction process difficult. Cements containing larger particles are obtained by grinding the grains agglomerated between the balls. For this reason, admixtures that prevent agglomeration by adsorbing to the particles cause a narrower particle size distribution.

#### 3.1.1. Flow Performance and Compressive Strength 

The PCE requirements and 1-, 7- and 28-day compressive strength values of the mixtures for the target flow value are given in Table 7.

When Table 7 is analyzed, it is observed that the PCE requirement for the target flow value increases with the use of GAs. This is thought to be due to the presence of finer particles due to the GA content (Figure 4). It was found that the early age strengths of the cements containing GAs were generally lower than the control mix. Each mortar mixture was prepared according to PCE requirements for target flow value. The PCE requirement for target flow value is generally increased in mixes containing GAs. This is thought to slow down the setting of these mixes, resulting in poor performance compared to the control mix [16].

When the 28-day mixtures were analyzed, it was determined that the GA content positively affected the compressive strength except for the mixtures containing DEA. In this context, it was determined that mixtures containing molasses, MEG and ethanol increased the compressive strength by 5–15%. It was determined that the highest strength performance was found in the mixtures containing molasses. Another reason for the high strength of the mixtures containing GAs compared to the control mixture is PSD. PSD is known to be an important parameter in the strength of cement [7,13,46,47]. Increasing the amount of fines decreases the proportion of non-hydrated particles. This has a positive effect on the strength of cement [7]. It reveals that the presence of GAs is effective in the progressive processes in hydration reactions. Considering that hardened cement pastes are porous materials, their porosity and pore structure are important factors affecting mechanical strength. Another critical factor in determining strength is the bulk strength of hardened cement pastes, which is governed primarily by the degree of hydration and the microstructure of cement hydrates.

#### 3.1.2. Dynamic Yield Stress, Viscosity Values and Structural Build-Up (A_thix_) Development of Mixtures

Dynamic yield stress and viscosity values of the cement paste mixtures produced within the scope of this study are shown in Table 8.

In order to clearly determine the effect of PCE usage rate, GA type and GA usage rate on the rheological properties of cement pastes, the effect of each parameter was evaluated separately.

When the rheological properties of mixtures without PCE are examined, it was seen that the rheological properties are negatively affected regardless of the type and dosage of GA. Assaad and Issa [15] reported that GAs negatively affect the fresh state and rheological properties of cementitious systems. When the particle size distribution (PSD) of the mixtures with GAs is examined, it is observed that the fine grain ratio increases as the GA dosage increases in cements with the same Blaine fineness value. It is thought that this situation may increase the PCE requirement and negatively affect the rheological properties.

When the effect of PCE usage rate on the dynamic yield stress and viscosity values of the mixtures was examined, decreases were detected in the dynamic yield and viscosity values of the mixtures due to the increase in the PCE usage rate, as expected in the control mixture without GAs. It is known that this situation is due to the electrostatic repulsion and steric repulsion forces acting between cement grains according to the colloidal dispersion stability theory [10,48]. However, it was understood that such behavior was not present in cement paste mixtures containing GAs. In these mixtures, it was determined that the dynamic yield stress and viscosity values generally decrease with an increase in PCE usage rate up to 0.08%, and these values may increase when PCE usage increases. Similarly, in a study conducted by Maybury et al. [49], it was determined that the mixtures exhibited shear thickening behavior when different additives with electrostatic and steric repulsion were added to the systems. It was reported by the researchers that this situation occurred due to the clustering of free polymer chains.

When the mixtures with 0.025% GA were examined, it was determined that the mixtures with the highest dynamic yield stress and viscosity values were the DEA and control mixtures, respectively. As the amount of admixture increased, it was understood that the mixture containing molasses and DEA generally had the highest value. Similarly, when the mixtures with 0.050% and 0.075% GA amounts were examined, regardless of the PCE usage rate, it was determined that the mixtures with the highest dynamic yield stress and viscosity values were the mixtures containing molasses and DEA. When the mixtures with 0.1% GA amount were examined, it was determined that the mixture with the highest dynamic yield stress and viscosity value was the mixture containing DEA. In the mixtures where the PCE usage amount was 0.08%, it was determined that the mixture containing molasses and MEG had the highest dynamic yield stress and viscosity value. In the mixture containing 0.16% PCE, it was determined that the highest dynamic yield stress and viscosity values were in the mixture containing DEA and MEG, respectively. As a result, it was determined that the additive containing molasses was generally the most effective on rheological parameters. As a result, it was determined that the additive containing molasses was generally the most effective on rheological parameters. Similarly, in a study conducted by Gao et al. [50], it was determined that the water requirement of the mixtures increased with the addition of molasses up to a certain dosage. However, it has been reported that adding molasses after a certain dosage does not increase the water requirement of the mixtures and even causes it to decrease. Researchers have stated that this situation is due to the development of cement particle fineness and specific surface with molasses dosage. It has been reported that the effect of using high amounts of molasses in reducing the water requirement is since molasses has a slight water-reducing effect in concrete [51,52].

A_thix_ values of the mixtures are shown in Table 3. When the effect of the PCE usage rate on the A_thix_ value of the mixtures was examined, as expected in the control mixture without GAs, decreases were detected in the A_thix_ values of the mixtures due to the increase in the PCE usage rate. This situation was similar to the trend measured in the rheological parameters of the mixtures. However, it was determined that such behavior was not present in the cement paste mixtures containing GAs and the structural recovery value of the mixtures generally increased with the increase in the PCE usage rate.

When the mixtures without PCE and with 0.025% and 0.050% GA amounts were examined, it was determined that the mixture with the highest A_thix_ value was the control mixture. It was determined that the structural build-up values of the mixtures changed depending on the type of GA with the increase in the PCE usage rate, and when the mixtures containing 0.08% PCE and 0.025% GA were examined, the mixture with the highest A_thix_ value was the mixture containing MEG. It was determined that the highest structural build-up rate belonged to the mixture using molasses when the GA usage rate was 0.050%. When the mixtures without PCE and with 0.075% GA were examined, it was determined that the mixture with the highest A_thix_ value was the mixture containing ethanol. In the mixture containing 0.08% and 0.16% PCE, the highest A_thix_ value was found to be in the mixture containing MEG. When the mixtures without PCE and with 0.1% GA were examined, it was determined that the mixture with the highest A_thix_ value was the mixture containing ethanol. In the mixture containing 0.08% PCE, it was determined that the highest A_thix_ value was in the mixture containing molasses. In the mixture containing 0.16% PCE, it was determined that the highest A_thix_ value was in the mixture containing MEG.

### 3.2. Evaluation of Analysis Results 

#### 3.2.1. Analysis of Signal-to-Noise (S/N) Ratios

Compressive strength values were measured experimentally for each combination of control factors with the experimental design. The data obtained were processed using Taguchi techniques and optimization of the measured grinding factors was achieved by signal-to-noise (S/N) ratios. The highest value of compressive strength is important to improving the quality of concrete. Therefore, the “higher is better” equation was used to calculate the S/N ratio. S/N ratios and mean values for the grinding parameters are shown in Table 9.

The effect of each control factor on the compressive strength was analyzed using a “S/N response table” and a “means table”. The results of the S/N and mean response of the compressive strength values are shown in Table 10.

**Figure 5:**
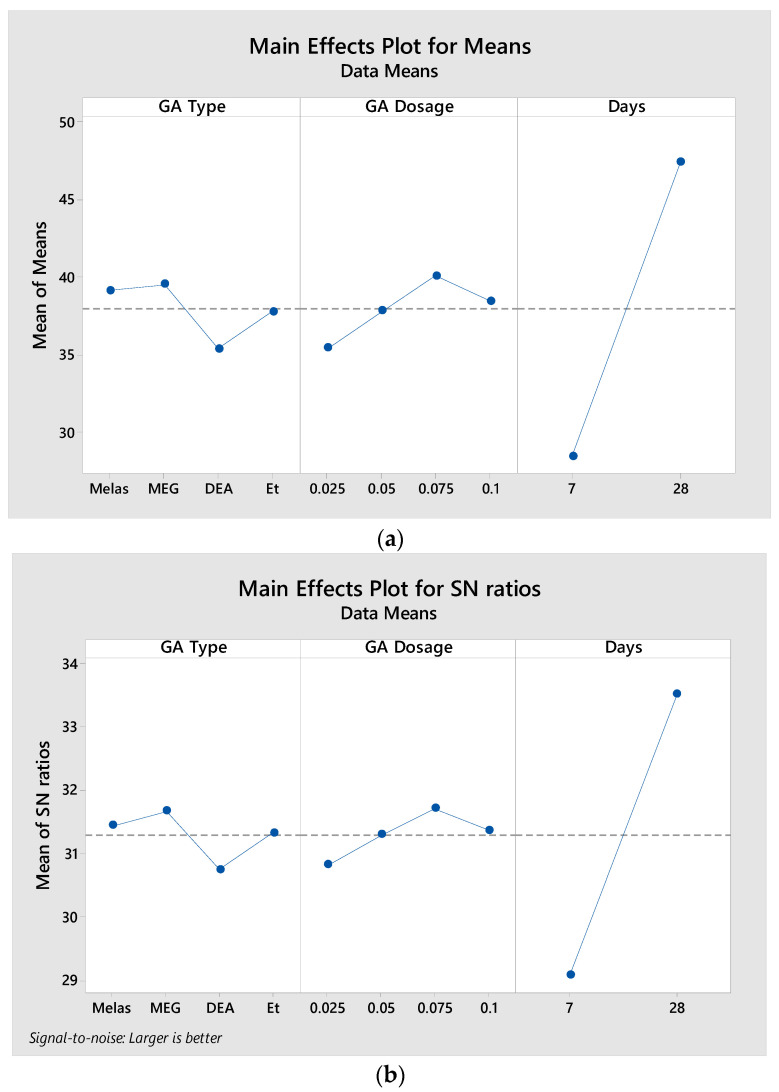
Influence degrees and significance graph of factor parameters for high compressive strength value (**a**) according to S/N ratio and (**b**) according to mean values.

This table, prepared using the Taguchi technique, shows the optimum levels of control factors for the highest compressive strength value. The level values of the control factors given in Table 10 are shown graphically in Figure 5. The optimum processing parameters of the control factors to achieve the highest compressive strength value can be easily determined from these graphs. The best level for each control factor was found according to the highest S/N ratio and the lowest mean value in the levels of that control factor. As a result, it was found that the highest compressive strength was obtained by curing MEG at 0.075 dosage for 28 days.

#### 3.2.2. The ANOVA Method

In this study, ANOVA was used to analyze the effects of GA type, dosage and curing time on compressive strength. The ANOVA results for the compressive strength value are shown in Table 11. This analysis was performed at 5% significance level and 95% confidence level. The significance of the control factors in ANOVA was determined by comparing the F values of each control factor. Moreover, if the P value of each control factor is less than 0.5, it means that the data are significant. The effect ratio in the last column of Table 11 shows the percentage value of each parameter contribution indicating the degree of impact on process performance. The percentage error of the ANOVA model was 2.38%.

#### 3.2.3. Estimation of Optimum Grinding Conditions by the Taguchi Method and Comparison with Experimental Results

For the Taguchi method, verification tests were performed at optimum and random levels of control factors. The results obtained are shown in Table 12. It is understood from the results that the predicted values and experimental data are quite close. Kıvak [25] reported that error values should be less than 20% for reliable statistical analysis. In this direction, it was understood that the data obtained were at an acceptable level. Therefore, the results obtained from the validation tests reflect successful optimization.

## 4. Conclusions

The results obtained from this study in which the effects of different types and dosages of GAs on grinding efficiency, rheological properties and compressive strength of cementitious systems were investigated are summarized below:

- It was determined that the highest and lowest grinding performance among the GAs was obtained when MEG and molasses admixtures were used, respectively.
- It was observed that the highest grinding performance, regardless of the type of GA, was obtained when 0.075% by mass of GA was used.
- It was found that the use of GAs increased the PCE requirement for the target spread value, had a negative effect on the early age strength but had a positive effect on the 28-day compressive strength.
- Decreases in the dynamic threshold shear stress, viscosity and A_thix_ values of the mixtures were measured due to the increase in the PCE utilization rate in the control mixture without GAs.
- The blends with the highest dynamic threshold shear stress and viscosity values were molasses 0.050–0.16% and MEG 0.1–0.08%, while the blends with the lowest values were control 0.16% and ethanol 0.075–0.16%, respectively.
- The mixture with the highest A_thix_ value was MEG 0.1–0.16%, while the mixture with the lowest value was Dea-0.1–0%.
- Optimum levels of control factors on compressive strength were determined using S/N ratios. It was found that the GA dosage was a more important parameter compared to the type of GA.
- The margin of error of the predictions compared to the experimental results was found to be acceptable.

## Figures and Tables

**Table 1:** Some chemical properties of clinker and gypsum [29].

	SiO_2_	Al_2_O_3_	Fe_2_O_3_	CaO	MgO	SO_3_ *	Na_2_O	K_2_O	Cl *	C_3_S	C_2_S	C_3_A	C_4_AF	Loss of Ignition
**Clinker**	21.52	5.43	3.31	65.38	1.04	0.38	0.48	0.54	0.01	56.51	19.06	8.79	10.07	0.52
**Gypsum**	4.98	1.21	0.83	28.94	0.83	39.67	0.25	0.19						

* According to TS EN 197-1, SO_3_ ≤ 3.5% and Cl^−^ ≤ 0.01.

**Table 2:** Some chemical properties of the used GAs and PCE.

Admixture Name	Molecular Weight (g/mol)	Alkaline Content (%) (Na_2_O)	Density (g/cm^3^)	Solid Content (%)	Chloride Content (%)	pH, 25 °C	Boiling Point	Number of OH Groups It Contains
Diethanol amine (DEA)	105.14	<10	1.097	50.0	<0.1	11.0	271	2
Monoethyleneglycol (MEG)	62.07	<10	1.112	50.0	<0.1	8.2	197	2
Ethanol	46.07	<10	0.789	50.0	<0.1	7.4	157	1
PCE	19,000	<10	1.06	32	<0.1	4.56	-	-

**Table 3:** Some chemical and physical properties of molasses used as GAs.

Name	Solid Content (%)	Sugar Content (%)	Raffinose (%)	pH 25 °C	Betaine (%)
Molasses	52.7	0.4	0.8	6.6	2.4

**Table 4:** Factor parameters and their levels.

Parameters	Symbol	Levels 1	Levels 2	Levels 3	Levels 4
GA Type	A	Molasses	MEG	DEA	Et
GA Dosage	B	0.025	0.05	0.075	0.1
Cure Duration	C	7	28	-	-

**Table 5:** Taguchi L16(4^2 2^1) orthogonal layout.

Experiment No.	Factor A	Factor B	Factor C
1	Molasses	0.025	7
2	Molasses	0.05	7
3	Molasses	0.075	28
4	Molasses	0.1	28
5	MEG	0.025	7
6	MEG	0.05	7
7	MEG	0.075	28
8	MEG	0.1	28
9	DEA	0.025	28
10	DEA	0.05	28
11	DEA	0.075	7
12	DEA	0.1	7
13	Ethanol	0.025	28
14	Ethanol	0.05	28
15	Ethanol	0.075	7
16	Ethanol	0.1	7

**Table 6:** Grinding efficiencies.

Cements	30 Min Specific Surface Area (cm^2^/g)	30 Min Relative Fineness Increase (%)	50 Min Specific Surface Area (cm^2^/g)	50 Min Relative Fineness Increase (%)	Blaine Fineness at the End of the Milling Process (cm^2^/g)	Time to Reach Target Blaine Fineness Value (min)	Energy Consumption (kWh/tone)	Relative Energy Efficiency Increase (%)
C	2180	-	3130	-	4100	96.00	47.200	-
0.025 Molasses	2325	6.65	3325	6.23	4050	85.48	42.960	8.98
0.05 Molasses	2260	3.67	3290	5.11	4100	84.41	42.448	10.07
0.075 Molasses	2390	9.63	3400	8.63	4095	83.55	42.036	10.94
0.1 Molasses	2250	3.21	3410	8.95	4100	82.30	41.436	12.21
0.025 MEG	2440	11.93	3450	10.22	4140	81.31	40.963	13.22
0.05 MEG	2500	14.68	3625	15.81	4170	80.24	40.450	14.30
0.075 MEG	2595	19.04	3660	16.93	4180	79.48	40.082	15.08
0.1 MEG	2520	15.60	3665	17.09	4150	80.26	40.457	14.29
0.025 DEA	2550	16.97	3570	14.06	4200	81.48	41.042	13.05
0.05 DEA	2350	7.80	3415	9.11	4140	80.83	40.730	13.71
0.075 DEA	2350	7.80	3470	10.86	4210	80.18	40.418	14.37
0.1 DEA	2360	8.26	3470	10.86	4200	82.83	41.688	11.68
0.025 Ethanol	2270	4.13	3310	5.75	4165	85.88	43.150	8.58
0.05 Ethanol	2390	9.63	3550	13.42	4180	81.94	41.262	12.58
0.075 Ethanol	2450	12.39	3605	15.18	4220	79.69	40.184	14.86
0.1 Ethanol	2385	9.40	3390	8.31	4210	82.81	41.678	11.70

**Table 7:** PCE requirements and compressive strength values of the mixtures.

Mixtures	Fresh State Unit Volume Weight (kg/m^3^)	PCE Requirement for Target Flow Value (%)	Compressive Strength (MPa)		
			1 Day	7 Day	28 Day
C	2.220	0.253	11.07	30.19	45.78
0.025 Molasses	2.318	0.250	11.81	28.37	47.86
0.05 Molasses	2.332	0.267	12.16	26.12	52.38
0.075 Molasses	2.281	0.241	10.80	25.64	50.80
0.1 Molasses	2.292	0.242	10.05	26.67	51.25
0.025 MEG	2.280	0.282	10.98	28.23	45.34
0.05 MEG	2.326	0.272	11.45	31.44	46.39
0.075 MEG	2.358	0.265	10.54	30.74	52.67
0.1 MEG	2.325	0.261	10.00	30.62	46.83
0.025 DEA	2.320	0.250	15.03	38.20	41.67
0.05 DEA	2.312	0.247	10.34	32.40	45.36
0.075 DEA	2.437	0.238	8.87	26.70	44.95
0.1 DEA	2.342	0.235	8.80	27.80	44.35
0.025 Ethanol	2.339	0.250	10.31	30.12	43.46
0.05 Ethanol	2.351	0.252	11.87	29.90	48.45
0.075 Ethanol	2.327	0.247	10.17	31.20	48.77
0.1 Ethanol	2.342	0.244	8.63	27.98	49.35

**Table 8:** Rheological properties of the mixtures.

Mixture	Rheological Parameters	PCE Dosage (%)		
		0	0.08	0.16
C	Dynamic yield stress (Pa)	16.70	10.57	6.12
	Apparent viscosity (Pa·s)	6.63	5.8	5.67
	A_thix_ (Pa/s)	0.56	0.63	0.5
0.025 Ethanol	Yield stress (Pa)	35	10.46	18.54
	Apparent viscosity (Pa·s)	5	5.33	5.09
	A_thix_ (Pa/s)	0.33	0.7	0.67
0.05 Ethanol	Yield stress (Pa)	37.31	23.11	29.25
	Apparent viscosity (Pa·s)	5.59	5.52	5.24
	A_thix_ (Pa/s)	0.48	0.57	0.79
0.075 Ethanol	Yield stress (Pa)	14.55	38.12	27.68
	Apparent viscosity (Pa·s)	7.22	6.25	4.82
	A_thix_ (Pa/s)	0.7	0.67	0.71
0.1 Ethanol	Yield stress (Pa)	42.89	29.38	31.95
	Apparent viscosity (Pa·s)	6.43	6.07	6.08
	A_thix_ (Pa/s)	0.72	0.66	0.92
0.025 MEG	Yield stress (Pa)	39.4	35.65	40.37
	Apparent viscosity (Pa·s)	6.3	6.2	6.55
	A_thix_ (Pa/s)	0.46	0.65	0.65
0.05 MEG	Yield stress (Pa)	47.95	38.48	40.35
	Apparent viscosity (Pa·s)	6.9	6.02	6.04
	A_thix_ (Pa/s)	0.54	0.65	0.76
0.075 MEG	Yield stress (Pa)	39.98	34.34	48.42
	Apparent viscosity (Pa·s)	6.17	8.27	7.07
	A_thix_ (Pa/s)	0.44	0.74	0.76
0.1 MEG	Yield stress (Pa)	37.39	16.24	47.59
	Apparent viscosity (Pa·s)	6.27	8.32	6.97
	A_thix_ (Pa/s)	0.56	1.2	1.59
0.025 DEA	Yield stress (Pa)	43.36	45.48	48.46
	Apparent viscosity (Pa·s)	5.9	5.8	6.74
	A_thix_ (Pa/s)	0.4	0.46	0.46
0.05 DEA	Yield stress (Pa)	40.36	43.69	51.68
	Apparent viscosity (Pa·s)	5.29	5.62	6.67
	A_thix_ (Pa/s)	0.35	0.39	0.55
0.075 DEA	Yield stress (Pa)	41.45	47.19	55.94
	Apparent viscosity (Pa·s)	5.54	6.38	7.23
	A_thix_ (Pa/s)	0.39	0.51	0.59
0.1 DEA	Yield stress (Pa)	57.39	50.94	58.38
	Apparent viscosity (Pa·s)	6.99	6.28	6.79
	A_thix_ (Pa/s)	0.31	0.42	0.35
0.025 Molasses	Yield stress (Pa)	43.1	46.26	61.78
	Apparent viscosity (Pa·s)	6.2	6.55	6.64
	A_thix_ (Pa/s)	0.48	0.61	0.75
0.05 Molasses	Yield stress (Pa)	50.58	50.89	63.51
	Apparent viscosity (Pa. s)	7.14	7.41	6.39
	A_thix_ (Pa/s)	0.51	0.68	0.72
0.075 Molasses	Yield stress (Pa)	57.7	53.51	49.39
	Apparent viscosity (Pa·s)	7.83	7.14	6.11
	A_thix_ (Pa/s)	0.56	0.72	1.2
0.1 Molasses	Yield stress (Pa)	48.12	57.85	38.14
	Apparent viscosity (Pa·s)	5.98	7.72	6.57
	A_thix_ (Pa/s)	0.42	0.74	0.73

**Table 9:** Experimental results, S/N ratios and mean values.

Experiment No.	Control Factors			Compressive Strength (MPa)	S/N Ratio for Compressive Strength	Means for Compressive Strength
	GA Type	GA Dosage	Days			
1	Molasses	0.025	7	28.37	29.06	28.37
2	Molasses	0.05	7	26.12	28.34	26.12
3	Molasses	0.075	28	50.8	34.12	50.8
4	Molasses	0.1	28	51.25	34.19	51.25
5	MEG	0.025	7	28.23	29.01	28.23
6	MEG	0.05	7	31.44	29.95	31.44
7	MEG	0.075	28	51.67	34.26	51.67
8	MEG	0.1	28	46.83	33.41	46.83
9	DEA	0.025	28	41.67	32.40	41.67
10	DEA	0.05	28	45.36	33.13	45.36
11	DEA	0.075	7	26.7	28.53	26.7
12	DEA	0.1	7	27.8	28.88	27.8
13	Et	0.025	28	43.46	32.76	43.46
14	Et	0.05	28	48.45	33.71	48.45
15	Et	0.075	7	31.2	29.88	31.2
16	Et	0.1	7	27.98	28.94	27.98

**Table 10:** Response table for S/N and significance for compressive strength values.

Response Table for Signal-to-Noise Ratios				Response Table for Means			
Level	GA Type	GA Dosage	Days	Level	GA Type	GA Dosage	Days
1	31.43	30.81	29.07	1	39.13	35.43	28.48
2	31.66	31.28	33.5	2	39.54	37.84	47.44
3	30.74	31.7		3	35.38	40.09	
4	31.32	31.36		4	37.77	38.46	
Delta	0.92	0.89	4.42	Delta	4.16	4.66	18.96
Rank	2	3	1	Rank	3	2	1

**Table 11:** ANOVA results for the energy consumed.

Source	Degre of Freedom (DoF)	Sum of Squares (SS)	Mean Square (MS)	F-Value	*p*-Value	Impact Rates (%)
GA Type	3	42.25	14.08	3.03	0.093168	2.71%
GA Dosage	3	44.82	14.94	3.22	0.082825	2.87%
Days	1	1437.36	1437.36	309.42	0	92.04%
Error	8	37.16	4.65			2.38%
Total	15	1561.59				100.00%

**Table 12:** Comparison of experimental results and Taguchi predictions.

	Taguchi Method		
	Experimental Result	Estimate	Error (%)
0.05 Molasses 28 Day	52.38	50.49	3.74
0.25 Ethanol 7 Day	29.90	27.77	7.67

## Data Availability

The original contributions presented in the study are included in the article, further inquiries can be directed to the corresponding author.
